# Characterization of *Nigella sativa* Meal (NSM) and the Effects on *In Vitro* Rumen Fermentation and Degradability

**DOI:** 10.3390/ani16071091

**Published:** 2026-04-02

**Authors:** Karina Natasya Juandita, Diky Ramdani, Iman Hernaman, Abdul Shakoor Chaudhry, Sukarman, Ki Ageng Sarwono

**Affiliations:** 1 Doctoral Program, Faculty of Animal Husbandry, Universitas Padjadjaran, Sumedang 45363, Indonesia; karina22009@mail.unpad.ac.id; 2 Department of Animal Production, Faculty of Animal Husbandry, Universitas Padjadjaran, Sumedang 45363, Indonesia; 3 Department of Animal Nutrition and Feed Technology, Faculty of Animal Husbandry, Universitas Padjadjaran, Sumedang 45363, Indonesia; iman.hernaman@unpad.ac.id; 4 School of Natural and Environmental Sciences, Newcastle University, Newcastle upon Tyne NE1 7RU, UK; abdul.chaudhry@newcastle.ac.uk; 5 Study Center for Applied Zoology, National Study and Innovation Agency (BRIN), Cibinong 16911, Indonesia; sukarman.2@brin.go.id (S.); kiagengsarwono@gmail.com (K.A.S.)

**Keywords:** enteric methane, functional feed, phytochemicals, rumen gas kinetics, ruminant nutrition

## Abstract

*Nigella sativa* meal (NSM) is a by-product of the oil extraction process with potential use as a functional feed ingredient in ruminant nutrition due to its rich bioactive compounds and nutrient content. Therefore, this replicated (*n* = 6) completely randomized design (CRD) study aimed to firstly characterize NSM for its the bioactive compounds by chromatography–mass spectrometry (GC–MS) and liquid chromatography–MS (LC–MS). The effects of its dietary inclusion at 0%, 5%, 7.5%, and 10% NSM on *in vitro* rumen fermentation, gas production, CH_4_ production, and rumen degradability were analyzed. Rumen fluid was collected orally from five sheep (body weight 20 ± 2 kg/head) and incubated with basal diets supplemented with pre-determined levels of NSM. The identified bioactive compounds identified included palmitic acid, oleic acid, linoleic acid, phenolic, flavonoid, thymoquinone, and saponin. The results showed that the dietary NSM supplementation in the diet significantly increased (*p* < 0.05) gas production at 18 and 48 h, NH_3_–N concentration, IVDMD (*In vitro* dry matter degradability), and IVOMD (*In vitro* organic matter degradability). However, there was no significant effect (*p* > 0.05) on gas production at 12 and 24 h, and CH_4_ production, pH, acetate, propionate, iso-butyrate, butyrate, iso-valerate, valerate, the acetate-to-propionate ratio (A:P), or total VFA concentration. Rumen fermentation was optimally modulated up to 10% without adverse effects on digestibility or CH_4_ production. In this context, NSM acted as a functional feed ingredient *in vitro*. Therefore, in vivo analyses are required to confirm the efficacy under practical feeding conditions.

## Simple Summary

The *Nigella sativa* L. oil extraction process generates a by-product known as *Nigella sativa* meal (NSM). This meal may be used as an affordable local feed to meet the growing challenge of limited feed availability to satisfy the nutritional requirements of sheep. This product has gained attention as a promising feed supplement due to its rich nutrient and bioactive contents such as palmitic, oleic, and linoleic acids, with phenolics, flavonoids, thymoquinone, and saponins. Moreover, including NSM at 10% of animal diets can enhance rumen gas production and improve nutrient digestibility, without increasing methane (CH_4_) production.

## 1. Introduction

In Indonesia, feeding practices in sheep farming systems are generally quite simple, with farmers still applying a traditional cut-and-carry method for supplying forages as the major feed. Ramdani et al. [1] reported that most of the high-quality grasslands in Indonesia were limited since many areas were converted to housing, industrial, and cropping lands. This condition presents challenges related to the nutritional requirements of sheep, particularly when using low-nutrient feed, such as corn stover, which is economical and readily available [2]. To compensate for the nutritional deficiencies and support optimal growth performance, the inclusion of suitable concentrates becomes necessary in sheep farming. However, the concentrates such as rice bran, palm kernel meal, and cassava pulp often contain a high fiber fraction. These products are typically formulated using fibrous agricultural by-products, but have led to a continual increase in the cost of feeding. Therefore, a high-quality and affordable alternative feed supplement is needed not only to increase feed efficiency, but also reduce production costs. Plants rich in nutrients and bioactive compounds represent a potential solution as a functional feed supplement and anthelmintic feed supplement. *Nigella sativa* L. seed is a part of the *Ranunculaceae* family and is used as a health-supporting herb in the Middle East, Far East, and West Asia [3]. The processing of *Nigella sativa* L. seeds produces *habbatussauda* essential oil. In Indonesia, approximately 144,817 kg/year of the seeds are used annually as raw material in the oil processing industry [4]. However, this industry also produces a by-product known as *Nigella sativa* meal (NSM). According to Obeidat [5], NSM represents 70–75% of the essential oil processing industry. This product serves as a functional feed supplement since the crude protein content is up to 36.8%. The presence of bioactive compounds in NSM may also contribute to enhanced growth performance, productivity, and immune function [4,5,6,7]. Previous studies reported that bioactive compounds such as thymoquinone (0.015%) and tannin (21.6%) had an effect on growth performance and nutrient efficiency of sheep [1,4].

Based on the description above, this study aimed to explore the bioactive components of NSM and evaluate the supplementation of NSM in the sheep diet on critical rumen degradability and fermentation parameters, including total gas production (tGP) and methane (CH_4_) gas production, pH levels, and individual volatile fatty acids (VFA). We hypothesized that the dietary inclusion of NSM would positively influence *in vitro* rumen fermentation, resulting in greater gas and VFA production, improved digestibility, and lower CH_4_ output due to its bioactive components.

## 2. Materials and Methods

### 2.1. Ethical Approval

The animal handling protocol in this study was approved by the Universitas Padjadjaran Study Ethics Commission (Approval number: 787/UN6.KEP/EC/2025; approved on 14 July 2025). This study was conducted following ARRIVE 2.0 guidelines for reporting animal studies, while rumen fluid oral collection was performed by trained veterinary personnel using procedures aimed to minimize animal stress as described in Rabee et al. [8]. Additionally, the animals were monitored throughout the sampling procedure and post-sampling period, with no adverse events reported.

### 2.2. Study Period and Location

The *in vitro* incubation, pH, CH_4_, IVDMD, IVOMD analyses were conducted from July to December 2025 at the Biotechnology Study and Testing Laboratory of the Faculty of Animal Husbandry, Universitas Padjadjaran. The NH_3_-N and individual VFA analyses were performed in November 2025 at the Central Laboratory of Universitas Padjadjaran.

### 2.3. Diet Preparation

The NSM by-product from black cumin (*Habbatussauda*) following its oil extraction was obtained from PT. Habbasyi Niaga Utama, Depok, Indonesia, and ground to pass through a 1 mm sieve in a hammer mill before being used as a diet ingredient. The commercial concentrate (Starter grade or SG2) was purchased from PT. Dilar Lintas Raya (Tasikmalaya, Indonesia), consisting of palm kernel meal (21%), cassava meal (15%), coffee husk (11%), rice bran (10.5%), copra meal (9%), molasses (8.5%), dried cassava (8.45%), cacao husk (6%), soybean meal (2%), rapeseed meal (1.5%), distillers’ dried grain with soluble (1%), wheat pollard (0.5%), mineral and vitamin premix (Lagantor F1 Customix, Kalbe Animal Health, 2%), lime (2%), corn gluten feed (1%), sodium bicarbonate (0.35%), and salt (0.2%). Furthermore, corn stover was prepared by chopping fresh corn stover (5–10 cm long) using a chopper machine (Tiger TPR900-2, Shanghai, China). The basal diet was prepared by mixing commercial concentrate and corn stover at a 60:40 ratio. Meanwhile, NSM formulation was supplemented with commercial concentrate. The details of the diet formulation and nutrient composition are provided in Table 1 and Table 2. The experimental diets were compared based on proximate analysis. The parameters analyzed included dry matter (DM), crude protein (CP), ether extract (EE), amylase-treated ash-free neutral detergent fiber (NDFom), and amylase-treated ash-free acid detergent fiber (ADFom).

### 2.4. Animals and Sampling

Approximately 100 mL of rumen fluid was collected from each of five non-pregnant and non-lactating Garut ewes. The donor animals with similar body weight (20 ± 2 kg/head) at approximately 8 months of age were housed on a slatted floor in the individual pens (1.0 m long × 1.0 m wide × 1.0 height). The average daily temperature and humidity during the experiment were 21.3 °C and 84.3% relative humidity (RH) (morning); 26.9 °C and 70.9% RH (afternoon), as well as 26.2 °C and 72.8% RH (evening). The donor animals received an oral anthelmintic (1 mL/head; Veta Bendazole^®^, PT. Sarana Veterinaria Jaya Abadi, South Tangerang, Indonesia), a vitamin B-complex injection (5 mL/head; B-Sanplex^®^, PT. Sanbe Farma, Bandung, Indonesia), and an antibiotic injection (2.5 mL/head; Limoxin-200 LA^®^, Interchemie Werken BV, Venray, The Netherlands) as part of routine health management three weeks before sampling. Subsequently, an experimental diet containing corn stover and commercial concentrate was offered to the samples in a ratio of 60:40. The experimental diet was formulated following the feeding and diet formulation following the feeding standard of Kearl [9] to meet the nutrient requirements of growing sheep with feed allowance set at approximately 3.5–4.0% of body weight on a DM intake basis (g/head/day) and targeting an average daily gain above 100 g/head/day. The diet was also divided into equal portions and offered twice daily at 08:00 and 16:00, and drinking water was offered *ad libitum* for 3 weeks before rumen fluid collection. Rumen fluid was collected orally using a stomach tube at a capacity of 500 mL connected to a vacuum pump (Shimadzu PC-268BIT, Tokyo, Japan) before morning feeding, with the initial 50 mL discarded to avoid saliva contamination, as described in Wang et al. [10]. The rumen fluid handling was conducted under strict anaerobic conditions. After the collection, rumen fluid was transferred into pre-warmed, insulated containers flushed with CO_2_ to minimize oxygen exposure and transported to the laboratory within 15 min. The rumen fluid was also filtered through four layers of muslin cloth and pooled under continuous CO_2_ flushing and maintained at 39 °C before incubation. During the preparation of the buffered inoculum and throughout the incubation setup, the CO_2_ was continuously flushed to maintain anaerobic conditions before the inoculum was dispensed into pre-warmed glass syringes and sealed for incubation. Moreover, the rumen fluid pH upon arrival was around 6.94.

### 2.5. In Vitro Incubation

The method used for rumen incubation *in vitro* followed Rahmatillah et al. [11] with several modifications. The buffered inoculum containing filtered rumen fluid was diluted with McDougall buffer [12], which was pre-flushed using CO_2_ gas and pre-warmed at 39 °C in a ratio of 2:1 (buffer: rumen fluid). The pH of each buffered inoculum was adjusted to around 7 ± 0.2 using HCl dropwise.

A 100 mL glass syringe (Fortuna Optima Poulten Graf, Wertheim, Germany) was filled with 200 ± 6 mg of feed DM. In addition, three blank syringes (without substrate) were prepared to correct for gas production from the buffered rumen fluid. Approximately 20 mL of the buffered RF inoculum were added to each glass syringe and incubated with syringes randomly positioned in a 39 °C water bath for 48 h with one incubation period. Total gas production (tGP) was recorded at 2 h intervals up to 24 h and then continued at 4 h intervals until 48 h of the incubation period. The warm water in the water bath was largely substituted with ice to effectively stop additional fermentation within the syringes. Subsequently, gas from each incubated syringe was transferred to another clean syringe with a stopcock for direct methane (CH_4_) gas analysis. The CH_4_ concentration in the produced gas was directly measured as a percentage (%) using a portable gas analyzer (Riken Keiki GX-2012 Confined Space Gas Monitor, RKI, Tokyo, Japan). Moreover, the CH_4_ production was subsequently calculated by multiplying total gas volume by the measured methane concentration and expressed as milliliters of CH_4_ per gram of incubated DM (mL CH_4_/g DM incubated) for statistical analysis following the method of Medjekal et al. [13]. All contents in each syringe, including inoculum and residue, were transferred to a previously weighed polyethylene tube with 20 mL capacity (PT Jayamas Medica Industri Tbk, Sidoarjo, Indonesia) for pH and *in vitro* dry matter degradability (IVDMD) analysis. The pH was measured directly using a calibrated pH meter (EUtech pH-700, Einhausen, Germany). Furthermore, the tubes were then centrifuged at 5000 rpm for 10 min, and the samples were prepared for volatile fatty acid (VFA) and ammonia (NH_3_-N) analysis, which were then frozen at −20 °C [14]. All residual particles remaining in the syringe were washed with water and placed into a suitable tube containing the residue. This undigested residue was dried at 60 °C using a drying oven for DMD determination, as described by Khan & Chaudry [15].

### 2.6. NH_3_-N Level Analysis

The ammonia (NH_3_-N) level was determined using the phenol-hypochlorite method using a spectrophotometer as described in Sagala et al. [14]. Approximately 100 µL of sample supernatant was added to a test tube for NH_3_-N analysis. In addition, 500 µL of a phenol solution (50 g L^−1^ phenol, 0.25 g L^−1^ sodium nitroprusside) and 500 µL NaOCl solution (16.9 mL L^−1^ NaOCl and 25 g L^−1^ NaOH) were mixed and added to the sample. The reaction tube was incubated in a 39 °C water bath for 20 min. The absorbance of the incubated samples was also measured using a UV-Vis spectrophotometer (PerkinElmer Lambda 35, Waltham, MA, USA) set at a wavelength of 630 nm.

### 2.7. Individual VFA Analysis

All samples were preserved using meta-phosphoric acid (Merck KGaA, Darmstadt, Germany) with a concentration of 200 g/L to the rumen fluid supernatant at a ratio of 1:4. The mixture was vortexed thoroughly and analyzed for individual VFA analysis using a method similar to that used by Rahmatillah et al. [11]. The samples were analyzed using gas chromatography–mass spectrometry (GC-MS) (Agilent 7890A, Santa Clara, CA, USA), a detector (MS Agilent 5977B GC-MS), and a column (Agilent, 123-3262 DB-FFAP) with a length of 60 m, a diameter of 0.325 mm, and a film thickness of 0.25 μm. The mobile phase was in the form of helium gas at a speed of 1 mL/min. A sample ratio and a 3 min delay time were set for injecting the syringe-collected free particles into the injector following Sagala et al. [14]. The injector temperature was set at 160 °C. The column temperature was maintained at 80 °C for 1 min, before increasing at a rate of 15 °C/min to 115 °C and held for 3 min. Subsequently, the temperature was raised at 3 °C/min to 130 °C, followed by a further increase at 15 °C/min to 230 °C for 3 min. The ratio of split injection was 15:1 as described in Rahmatillah et al. [11]. Individual VFA values were identified and measured based on the value of the sample peak area compared to the standard single peak area (Volatile free acid mix; Supelco. CRM46975, Bellefonte, PA, USA) containing 10.00 mM acetic acid, propionic acid, isobutyric acid, butyric acid, isovaleric acid, and valeric acid.

### 2.8. Chemical Properties Analysis

Chemical analyses were conducted to characterize the feed ingredients and experimental diet described in Table 1 and Table 2. Feed ingredients, such as NSM, commercial concentrate, and corn stover were collected from storage bins to determine dry matter (DM) content. For each type of feed, samples were collected from various parts (top, middle, and bottom) of the representative storage bins. Each feed ingredient was dried in an oven at 58 °C for 48 h. The dried samples were ground in a disk mill and passed through a 1 mm screen before being analyzed for various nutrient compositions. The following AOAC [16] methods used for DM, ash, CP, and ether extract were 934.01, 942.05, 984.13, and 920.39, respectively. The neutral detergent fibers (NDFom, without decalin and α-amylase) and acid detergent fibers (ADFom) were analyzed following the method of Van Soes et al. [17]. Metabolizable energy (ME) was calculated using the procedure of Menke and Steingass, as described by Ramdani et al. [1].

The calcium (Ca) and phosphorus (P) contents of NSM were analyzed using the atomic absorption spectrophotometer (AAS) and UV-Vis spectrophotometer (PerkinElmer Lambda 35, USA) following Yang et al. [18]. Phyto-components of NSM were also identified using a GC-MS and LC-MS detection system. The Gas Chromatography–Mass Spectrometry (GC-MS) analysis was accomplished using Agilent 7890A (Agilent Technologies, USA), a detector (MS Agilent 5977B GC-MS) and direct capillary column TG–5MS (Thermo Fisher Scientific, Waltham, MA, USA) (30 m × 0.25 mm × 0.25 m film thickness). The GC oven temperature was set at 50 °C, gradually increasing at a rate of 5 °C per minute to 230 °C and maintained for 2 min. Furthermore, the temperature was increased more rapidly (30 °C per minute) to 290 °C, and maintained for another 2 min. The temperatures of the injector and MS transfer line were maintained at 250 °C and 260 °C, respectively. Helium was used as the carrier gas with a constant flow rate of 1 mL/minute. A 3 min solvent delay was applied, and 1 µL of each diluted sample was automatically injected using an AS1300 autosampler in split mode. Mass spectra were also recorded under electron impact ionization (70 eV) over the *m*/*z* range 40–1000 in full scan mode, with the ion source temperature set at 200 °C. These compounds were identified by comparing the retention times and mass spectra with data contained in the WILEY 09 and NIST 11 mass spectrum libraries. In addition, chromatographic separation of phytochemical components was achieved using a Poroshell 120 EC-C18 column from Agilent Technologies (USA), with dimensions of 100 mm × 4.6 mm I.D., and particle size 2.7 µm, under a gradient elution program applied at a flow rate of 0.4 mL/min. Phytochemical analysis of the diluted extract solutions was performed using liquid chromatography–tandem mass spectrometry (LC–MS/MS), and the analysis was carried out on an LCMS-8060RX system (Shimadzu, Japan). Moreover, most nutrient compositions were expressed in %DM, excluding ME level (MJ/kg DM) and DM (% fresh sample).

### 2.9. Statistical Analysis

This study used a completely randomized design (CRD) with four treatments and six replications (*n* = 6). Each replication represented an individual glass syringe used as the experimental unit. The residual data were examined for normality by Shapiro–Wilk test *p* > 0.05. All data sets were then submitted for statistical analysis using the General Linear Model in IBM SPSS 26.0 software (SPSS Institute, Chicago, IL, USA) as a completely randomized design. The Analysis of Variance (ANOVA) for all studied traits was conducted at *p* ≤ 0.05 and the differences between means or treatments were analyzed by the Tukey test at *p* ≤ 0.05 to identify differences among treatments.

## 3. Results

### 3.1. Bioactive Compounds of NSM

The chemical composition of NSM was characterized using complementary GC–MS and LC–MS analyses (Table 3 and Table 4). The GC–MS analysis predominantly identified non-polar lipid compounds, mainly fatty acids and derivatives, including palmitic, lauric, oleic, linoleic, and conjugated linoleic acids (CLA). In contrast, the LC–MS analysis showed the presence of polar and semi-polar bioactive compounds, with triterpenoid saponin derivatives as the dominant constituents, followed by flavonoid, phenolic acid, and quinone-related compounds. The combination of the analytical methods provided a comprehensive chemical profile of NSM, including lipid-based and phytochemical bioactive fractions.

### 3.2. Effect of NSM Supplementation on Gas Production and Rumen Fermentation Profile

**Figure 1:**
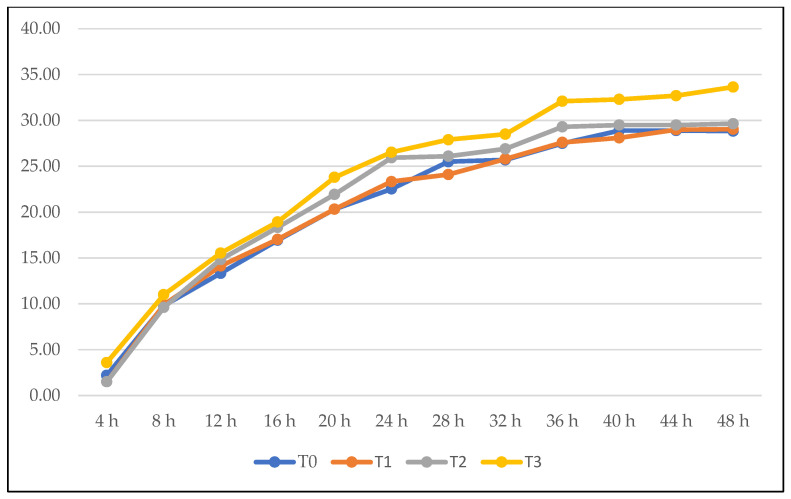
Total gas production (mL) during 48 h of incubation period.

Table 5 shows that varying inclusion levels of NSM in sheep diet have significant effects on tGP at 18 h and 48 h, NH_3_-N level, IVDMD and IVOMD (*p* < 0.05). At 18 h of incubation, tGP varied significantly among treatments, which was greatest in T3 (23.4 mL) and exceeded that of the control (T0; 20.4 mL). Meanwhile, T1 and T2 led to an intermediate gas production with no significant differences from T0 and T3 (*p* > 0.05). At the end of the 48 h of incubation, the lowest gas volume was observed in T0 (28.8 mL). Conversely, T1 (29.4 mL), T2 (29.6 mL), and T3 (33.6 mL) produced significantly higher gas volumes than T0. Figure 1 shows that total gas production increased progressively with incubation time in all treatments. A typical fermentation pattern was reported with a rapid increase during the early phase (4–24 h) followed by a gradual plateau toward 48 h. The NSM-supplemented treatments (T1–T3) generally produced higher total gas than the control (T0), with the highest values consistently observed in T3. Treatment differences were reported after 20 h which persisted until 48 h, with tGP increasing from the control to the highest NSM level. The NSM up to 10% in the diet could increase the NH_3_-N level significantly (*p* < 0.05) compared to the control (T0). The supplementation of NSM in the diet enhanced IVDMD compared to the control diet (T0). IVDMD increased from 50.0% in T0 to above 57% in all NSM-supplemented treatments, with no significant differences observed among the supplemented groups. T0 showed the lowest IVOMD (51.5%), while all NSM-supplemented treatments achieved similarly higher digestibility values of 58.6% to 60.1%. However, a non-significant effect (*p* > 0.05, Table 5) could be seen on tGP 12 and 24 h, CH_4_ (%), CH_4_ (mL), pH, acetate, propionate, iso-butyrate, butyrate, iso-valerate, valerate, A:P, and total VFA.

## 4. Discussion

The bioactive compound profiling of NSM using GC–MS and LC–MS shows the complex nature of this by-product and supports its potential use as a functional feed ingredient of sheep. The GC–MS analysis reported that NSM retained a substantial lipid fraction even after oil extraction. The lipid fraction is primarily composed of fatty acids and their derivatives, including palmitic acid, oleic acid, linoleic acid, and conjugated linoleic acids (CLA). Fatty acids are known to interact with rumen microorganisms and influence fermentation processes [19]. Polyunsaturated fatty acids like linoleic acid could serve as alternative hydrogen sinks during ruminal biohydrogenation, which may help reduce hydrogen availability for CH_4_ formation [20]. Medium-chain saturated fatty acids, including lauric and myristic acids, have been widely reported to suppress rumen protozoa. Therefore, the lipid fraction of NSM contributes to improved energy utilization and lower CH_4_ losses. Cisuayuni et al. [21] reported that lauric acid was an effective agent for mitigating methanogenesis in the rumen.

The LC-MS analysis showed that NSM was also rich in bioactive phytochemicals, with triterpenoid saponin derivatives identified as the most abundant polar compounds, followed by flavonoid, phenolic acid, and thymoquinone-related quinone. According to Joch et al. [22], saponin can disrupt protozoal cell membranes, leading to a reduction in protozoal populations and indirectly limiting methanogenic activity due to the close association between protozoa and methanogens. Flavonoids and phenolic acids add further functional value through their antimicrobial and antioxidant properties and have been associated with shifts in rumen fermentation toward higher propionate production to improve energy efficiency in ruminants [23]. Kim et al. [24] also reported that flavonoid-rich plant extracts reduced CH_4_ gas in the rumen. The detection of thymoquinone-related compounds shows that key bioactive compounds of *Nigella sativa* remain present in the meal fraction, even after oil extraction, and may contribute to the overall biological activity of NSM. Retnani et al. [4] found that NSM contained low amounts of thymoquinone, which exhibits significant antibacterial activity against a wide range of pathogenic bacteria, fungi, and protozoa, which may contribute to enhanced microbial protein [25].

In this study, cumulative gas production reported a clear time-dependent response to NSM supplementation (Table 5). The non-significant differences during the early incubation period (12 and 24 h) showed that NSM did not interfere with initial microbial colonization or the fermentation of rapidly degradable substrates, controlling early gas kinetics in *in vitro* systems [26]. The significant increases observed at 18 h and 48 h suggested that the effects of NSM became more pronounced during the active and later stages of fermentation due to increased microbial degradation of structural carbohydrates. The greater cumulative gas production in supplemented treatments, particularly at the highest inclusion level, reflects enhanced fermentability and microbial efficiency. This response may be connected to the presence of bioactive fatty acids and phenolic compounds identified in NSM through GC–MS and LC–MS analyses, which have been reported to modulate rumen microbial activity and improve substrate accessibility [26,27]. Wang et al. [10] also reported that gas production was a key indicator of feed fermentability and rumen microbial activity. NSM is rich in crude protein, potentially providing an energy source for rumen microbes, which leads to an increased gas production as a by-product of microbial metabolism [28,29].

Despite the increase in cumulative gas production, the CH_4_ production was not significantly affected by the NSM supplementation (*p* > 0.05). The emission observed in this study ranged from 12.7 to 21.2%. In contrast, a previous study reported that the addition of NSM reduced CH_4_ production [13]. CH_4_ produced in this study was generally lower than the level reported in the previous study, which documented values up to 17.2% [11]. In terms of absolute volume, the CH_4_ production ranged from 4.34 to 6.32 (mL/200 mg DM), again lower than previously reported values, where the NSM supplementation was associated with CH_4_ production reaching 12.19 mL/300 mg DM [6]. The NSM contains saponin, which is often associated with anti-methanogenic activity through protozoal suppression, but CH_4_ production was not affected in the present study. This may be explained by the relatively low effective saponin level and microbial compensation mechanisms, where reductions in protozoa-associated methanogens are offset by increased activity of free-living types, maintaining interspecies hydrogen transfer and overall CH_4_ production [6].

Previous studies showed that fatty acids such as lauric, oleic, and linoleic acids suppressed methanogenesis through direct inhibition of methanogenic archaea and protozoa, but the effects were strongly dose- and matrix-dependent [29,30,31]. Medjekal et al. [13] reported that oleic and linoleic acids were the two major fatty acids of *Nigella sativa*, which are responsible for anti-methanogenic activity. Phenolic compounds in NSM may reduce CH_4_ formation by altering hydrogen availability and microbial population structure, yet moderate concentrations often result in variable responses [32]. Therefore, this study suggests that NSM primarily enhanced overall fermentation efficiency rather than selectively redirecting hydrogen toward reduced CH_4_ synthesis.

In the current study, rumen pH was not affected by the level of NSM. However, the results showed that NSM could maintain rumen pH stability. The rumen pH reflects the fermentation conditions in the rumen [14]. In the current study, rumen pH ranged between 6.52 and 6.74. Several studies reported the normal rumen pH ranged from 6.40 to 6.81 [6,33]. The rumen pH is the result of acid production, salivary buffers, and the feed’s own buffers [13]. Rumen pH remained stable and within the optimal physiological range across all treatments, showing that NSM supplementation did not disrupt ruminal acid–base homeostasis. Maintaining a stable pH is essential for sustaining fibrolytic microbial populations and efficient fiber degradation [34]. The absence of pH changes despite increased cumulative gas production suggested that fermentation end products were generated in a balanced manner. Bioactive compounds in NSM, particularly phenolics, may regulate microbial growth rates and limit the fermentation of soluble carbohydrates, thereby preventing excessive acid accumulation [35].

The NH_3_-N levels in this study ranged from 7.39 to 10.33 mM. McDonald et al. [36] reported that the optimum amount of ammonia in the rumen was 6–21 mM. The fermented feed in the rumen in this study produced normal amounts of NH_3_-N levels. This describes the degradation products of feed protein and or NPN since excessive amounts show inefficiency [37]. Widyarini et al. [6] reported that the higher protein content in NSM was associated with higher concentration of NH_3_-N. Increased NH_3_-N concentration may reflect protein degradation by microbes. Microbes degrade more than 60% of protein in the rumen into amino acids, peptides, and NH_3_-N. The presence of NH_3_-N is intended to be used for microbial protein synthesis [14].

The NSM supplementation in the diet enhanced the extent of substrate degradation. These improvements are consistent with the greater cumulative gas production observed during later incubation periods and confirm that NSM improved fermentation efficiency rather than merely increasing gas output. NSM has many benefits in increasing degradability due to increased nutrients and bioactive compounds. Previous studies reported that fatty acids, including palmitic and oleic acids, increased microbial enzymatic activity and fiber accessibility. Oleic acid in proportions resembling those found in the mixed rumen bacteria increases fiber degradation in the rumen [38]. Phenolic compounds may also selectively inhibit less efficient microbial groups, allowing more effective fibrolytic communities to dominate and increase degradability [39]. Crude protein in NSM potentially stimulates the growth of rumen microorganisms and enhances feed degradability. An increase in rumen microbial population enhances NH_3_-N utilization, fiber degradation, and microbial protein synthesis. These processes increase feed breakdown and ultimately improve degradability [32].

In the current study, acetate, propionate, and total VFA concentrations tended to be higher in the supplemented treatments. According to Widyarini et al. [6], *Nigella sativa* did not affect the total VFA production and the proportion of acetate, propionate, and butyrate. VFA contributes about 70% of the energy needs of the livestock, and then it can be absorbed from the rumen wall [13]. In this study, total VFA concentrations ranged from 32.0 mM (T0) to 42.0 mM (T3) since the NSM supplementation in the diet did not adversely affect rumen fermentation.

This study has several limitations that should be considered when interpreting the results. First, the experiment was conducted using an *in vitro* rumen fermentation system, which may not fully represent the complex physiological conditions occurring in the in vivo study. Therefore, the responses observed in this study may differ under practical feeding conditions. In addition, rumen fluid used as inoculum was collected from only five donor sheep, which may represent a limitation of this study. Further in vivo study is required to confirm the efficacy of dietary NSM under practical feeding conditions.

## 5. Conclusions

In conclusion, NSM showed a dual type of bioactive compounds, combining lipid-based fatty acids with polar phytochemicals dominated by triterpenoid saponins. The compounds were related to the enhanced rumen fermentation efficiency, as shown by increased tGP, NH_3_-N, and improved *in vitro* digestibility. Although methane (CH_4_) production was not significantly affected, a tendency toward reduced methane production was observed in some treatments, suggesting potential antimethanogenic activity.

## Figures and Tables

**Table 1:** Proximate analysis of NSM.

Nutrient Compositions	Level
Dry Matter (% fresh sample)	96.1 ± 3.55
Metabolizable Energy (MJ/kg DM)	6.55 ± 0.13
Ash (% DM)	6.94 ± 0.22
Organic matter (% DM)	93.0 ± 0.22
Crude protein (% DM)	35.9 ± 5.91
Ether extract (% DM)	5.79 ± 0.34
Acid detergent fibers (% DM)	15.2 ± 0.50
Neutral detergent fibers (% DM)	25.2 ± 0.88
Calcium (% DM)	0.20 ± 0.04
Phosphorus (% DM)	1.68 ± 0.32

Values represent mean ± SD of triplicate analyses (*n* = 3).

**Table 2:** Formulation and nutrient compositions of the experimental diet.

Ingredients	Experimental Diet			
	T0	T1	T2	T3
Corn Stover (%)	60.0	60.0	60.0	60.0
Commercial Concentrate (%)	40.0	35.0	32.5	30.0
*Nigella sativa* Meal (%)	0.00	5.00	7.50	10.0
Nutrient Compositions:				
DM (% fresh sample)	52.5	53.1	53.4	53.7
ME (MJ/kg DM)	4.97	4.97	4.97	4.97
Ash (% DM)	9.39	9.23	9.14	9.06
CP (% DM)	11.6	12.6	13.2	13.7
EE (% DM)	4.15	4.13	4.12	4.11
ADFom (% DM)	35.9	35.1	34.7	34.3
NDFom (% DM)	58.9	57.7	57.1	56.6

DM = dry matter, ME = metabolizable energy, CP = crude protein, EE = ether extract, NDFom = amylase-treated, ash-free neutral detergent fiber, ADFom = amylase-treated, ash-free acid detergent fiber, T0 = control diet with no NSM; T1, T2 and T3 were diets with 5, 7.5 and 10% NSM respectively.

**Table 3:** Identification of chemical compounds in NSM by GC-MS.

No	Retention Time (min)	Compound Name	Chemical Class	Area (%)
1	14.25–14.60	1-Nonanol	Alcohol	4.90
2	15.82–16.19	2,4-Decadienal	Aldehyde	7.86
3	17.88–18.05	2-Undecenal	Aldehyde	8.16
4	19.72–20.16	4-Oxononanal	Aldehyde	5.90
5	20.80–21.16	Hexanoic acid (C6:0)	Saturated fatty acid	6.14
6	24.40–24.51	cis-4,5-Epoxy-(E)-2-decenal	Epoxy-aldehyde	5.99
7	25.94–26.18	Octanoic acid (C8:0)	Saturated fatty acid	6.14
8	34.46–34.56	Oleic acid methyl ester	Fatty acid ester	5.50
9	35.14–35.25	Dodecanoic acid (Lauric acid, C12:0)	Saturated fatty acid	6.00
10	35.31–35.40	Linoleic acid methyl ester	Fatty acid ester	6.70
11	36.09–36.18	CLA ethyl ester	PUFA ester	6.70
12	39.21–39.32	Tetradecanoic acid (C14:0)	Saturated fatty acid	7.07
13	41.97–42.10	Glycidyl palmitate	Fatty acid derivative	7.38
14	43.04–43.70	n-Hexadecanoic acid (Palmitic acid, C16:0)	Saturated fatty acid	10.0
15	45.98–46.18	Oleic acid oxiranylmethyl ester	Unsaturated FA derivative	8.22
16	46.66–46.97	Octadecanoic acid (Stearic acid, C18:0)	Saturated fatty acid	6.08
17	46.99–47.46	cis-13-Octadecenoic acid (Oleic acid, C18:1)	MUFA	8.40
18	47.81–48.40	Conjugated linoleic acid (CLA)	PUFA	9.51
19	48.69–49.91	Linoleic acid (C18:2)	PUFA	8.95
20	51.58–51.69	Diepoxyhexadecane	Epoxy compound	4.62

Area (%) represents relative peak area expressed as a percentage of the total detected area (sum = 100%).

**Table 4:** Tentatively identified compounds in NSM by LC–MS.

RT (min)	*m*/*z*	Ion Mode	Tentative Compound	Chemical Class
3.53–5.68	279–313	[M − H]^−^	Phenolic acid derivatives	Phenolic
9.51–13.90	285–342	[M + H]^+^	Flavonoid aglycones & glycosides	Flavonoid
11.38–16.72	203–205	[M + H]^+^	Thymoquinone-related compounds	Quinone
12.95–17.67	559–587	[M + H]^+^/[M − H]^−^	Triterpenoid saponin derivatives	Saponin

RT = Retention time (min), *m*/*z* = mass-to-charge ratio, LC–MS = liquid chromatography–mass spectrometry, [M + H]^+^ and [M − H]^−^ = protonated and deprotonated molecular ions; compounds were tentatively identified using accurate mass (<5 ppm), fragmentation patterns, and literature comparison; final confirmation requires isolation and NMR analysis.

**Table 5:** *In vitro* gas production and rumen fermentation profiles.

Parameters	Treatments				SEM	*p*-Value
	T0	T1	T2	T3		
Cumulative gas production:						
tGP 12 h (mL)	13.3	14.2	14.7	15.5	0.76	0.279
tGP 18 h (mL)	20.4 ^ab^	19.5 ^a^	22.2 ^ab^	23.4 ^b^	0.96	0.048
tGP 24 h (mL)	22.5	23.8	25.9	26.5	1.03	0.075
tGP 48 h (mL)	28.8 ^a^	29.4 ^a^	29.6 ^a^	33.6 ^b^	1.01	0.038
CH_4_ (%)	21.2	16.2	16.8	12.7	2.34	0.121
CH_4_ (mL/200 mg DM)	6.32	4.86	5.08	4.34	0.82	0.414
Rumen fermentation profiles *in vitro*:						
pH	6.52	6.52	6.66	6.74	0.05	0.279
NH_3_-N (mM)	8.57 ^ab^	7.39 ^a^	7.96 ^ab^	10.33 ^b^	0.60	0.042
IVDMD	50.0 ^a^	57.5 ^b^	58.5 ^b^	59.4 ^b^	1.50	0.004
IVOMD	51.5 ^a^	58.6 ^b^	59.7 ^b^	60.1 ^b^	1.56	0.006
Acetate (mM)	21.9	22.3	28.4	29.3	2.88	0.149
Propionate (mM)	5.79	5.80	7.55	7.17	0.64	0.111
Iso-butyrate (mM)	1.08	0.72	2.90	1.42	0.52	0.206
Butyrate (mM)	0.41	1.45	2.30	1.19	0.49	0.103
Iso-valerate (mM)	0.25	0.56	0.45	0.49	0.12	0.733
Valerate (mM)	0.33	1.71	0.39	0.34	0.25	0.085
A:P	3.76	3.94	3.88	4.13	0.41	0.967
Total VFAs (mM)	32.0	32.4	42.0	39.9	3.63	0.125

tGP = total gas production, IVDMD = *In vitro* dry matter degradability, IVOMD = *In vitro* organic matter digestibility, A:P = acetate: propionate, T0 = control diet with no NSM; T1, T2 and T3 were diets with 5, 7.5 and 10% NSM respectively; ^a,b^ means with different superscripts showed a significant difference (*p* < 0.05) according to Tukey’s HSD test; SEM = standard error of the mean.

## Data Availability

The data that support the results in this study are available from the corresponding author upon reasonable request.

## Abbreviations

The following abbreviations are used in this manuscript:

ADFom	Amylase-treated, ash-free acid detergent fiber
CP	Crude protein
DM	Dry matter
EE	Ether extract
IVDMD	*In vitro* dry matter degradability
IVOMD	*In vitro* organic matter degradability
NDFom	Amylase-treated, ash-free neutral detergent fiber
NSM	*Nigella sativa* meal
tGP	Total gas production
VFA	Volatile fatty acid
